# Sudden cardiac death—update

**DOI:** 10.1007/s00414-020-02481-z

**Published:** 2020-12-21

**Authors:** P. Markwerth, T Bajanowski, I. Tzimas, R. Dettmeyer

**Affiliations:** 1 Institute for Forensic Medicine, University Hospital Essen, Hufelandstr. 55, 45122 Essen, Germany; 2 Institute for Forensic Medicine, University Hospital Gießen, Giessen, Germany

**Keywords:** Sudden cardiac death (SCD), Forensic aspects, Acute death, Death in the young, Common cause of death

## Abstract

Sudden cardiac death (SCD) is one of the most common causes of death worldwide with a higher frequency especially in the young. Therefore, SCD is represented frequently in forensic autopsy practice, whereupon pathological findings in the heart can explain acute death. These pathological changes may not only include myocardial infarction, coronary thrombosis, or all forms of myocarditis/endocarditis but also rare diseases such as hereditary structural or arrythmogenic anomalies, lesions of the cardiac conduction system, or primary cardiac tumours.

## Introduction

Sudden and unexpected deaths are the most important and common differential diagnosis of death from non-natural causes in forensic medicine. Therefore, these deaths are a considerable part of the sectional material of forensic medicine in developed countries [1]. Every year, about 350,000 people die suddenly and unexpectedly in Europe and between 300,000 and 400,000 in the USA. Thus, these deaths are as frequent as deaths from breast cancer, lung cancer and colorectal cancer [2, 3]. The majority of sudden deaths is caused by cardiac alterations and known as ‘sudden cardiac death’ (SCD).

## Definition

The concept of sudden and unexpected death is not without problems, since the term ‘unexpected’ implies a death occurring out of apparent health or after banal, short-term symptoms of illness. Therefore, knowledge about the state of health of the person concerned is essential for the assessment, but often neither relatives nor treating physicians have such comprehensive knowledge about the person who died. Thus, doctors repeatedly make statements to the police such as ‘the deceased 85-year-old patient was in perfect health’, while serious circulatory disorder based on high-grade general atherosclerosis is not mentioned. In addition, clear medical misdiagnoses may occur, if relevant cardiac symptoms are misinterpreted [1].

Simultaneously, the temporal evaluation ‘suddenly’ is often a highly subjective assessment without a fixed definition. The external circumstances of the dying process, the finding situation and again the available level of information greatly influence such an evaluation. With regard to the SCD, the process of dying (if known) can be used as a criterion by measuring the period from the onset of the first cardiac symptoms to the clinically determined death.

According to Hering [4], the duration of death at SCD should be a few seconds, whereas an older WHO proposal states 1 h [5]. A newer publication of Semsarian et al. defined the SCD as a death within 1 h after the first symptoms of a heart disease unknown up to that time [6]. Since many of the sudden cardiac death cases occur outside hospitals and unobserved by witnesses, the concept of sudden death is problematic in its application. In many cases, neither reliable information about the beginning of symptoms nor about the time of death is available.

## Etiology

With regard to the etiology of the SCD, it is advisable to consider different stages of life in a differentiated way. SCD represents a very rare event in infancy, childhood and adolescence but typically leads to death within a few minutes [7]. For the USA, frequencies for the infantile SCD between 0.6 and 6.2 cases per 100,000 patients have been published [8]. About 25% of these cases occur during physical activities, e.g. sports [9]. The most common causes are malformations of the heart and/or the large vessels or genetic cardiac arrhythmias. In younger and middle-aged adults, however, myocarditis, cardiomyopathies and heart rhythm disturbances (for example, also in connection with a mitral valve prolapse syndrome) are more frequent causes of death [6, 10]. Here, a study performed in New Zealand and Australia showed a frequency of 3.2 cases per 100,000 persons in the age range 31 to 35 years, with a total of 72% of boys or men affected in the age range up to 35 years [11].

**Fig. 1:**
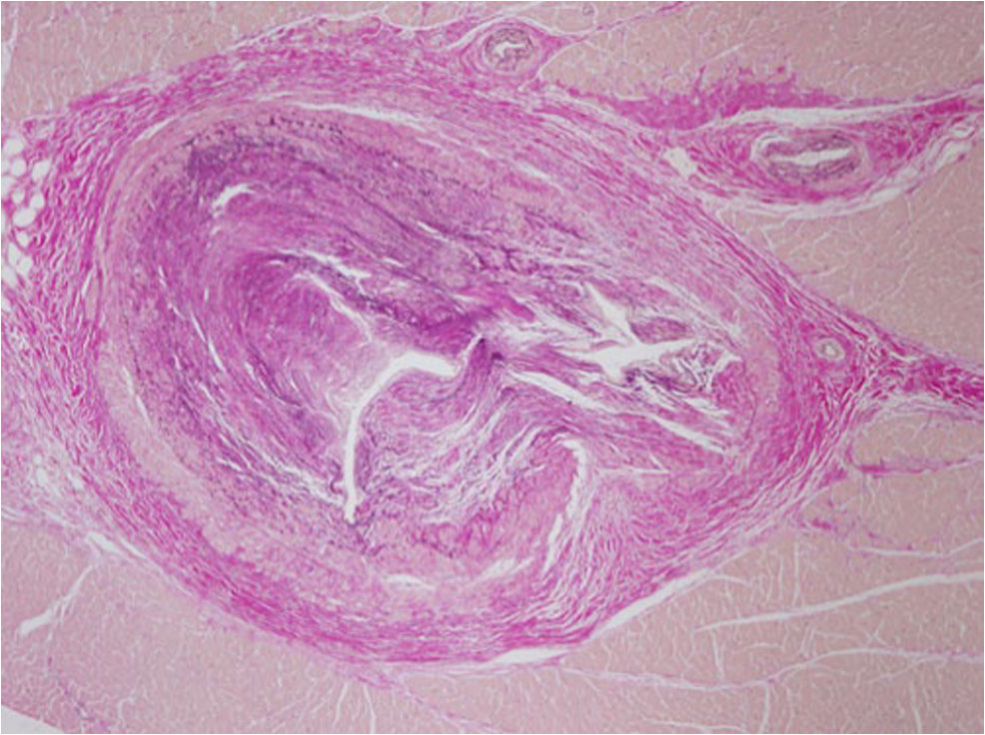
Highly stenosing fibromuscular dysplasia of the AV nodal artery with broad connective tissue thickening of the vessel wall and partial destruction of the lamina elastica interna without inflammatory component (Elastica van Gieson staining (EvG), × 40)

**Fig. 2:**
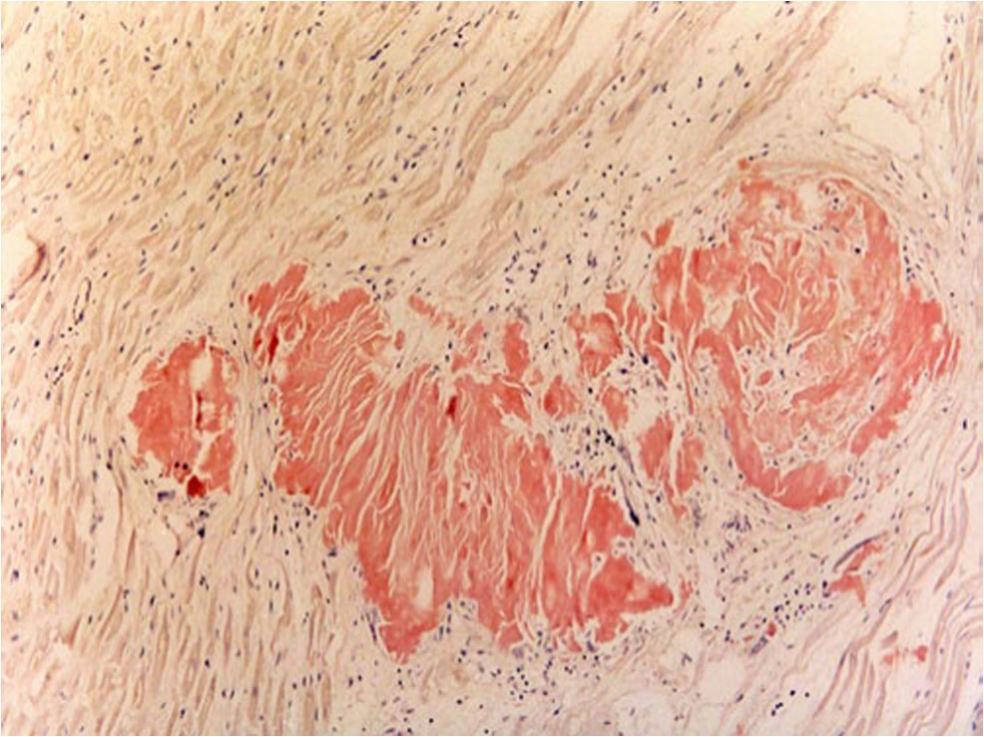
Congo red positive amyloid plaques found in the myocardium with surrounding low lymphomonocyte-infused fibrosis (Congo red staining, × 200)

In older people, circulatory disorders of the myocardium up to and including acute myocardial infarction predominate. SCD is frequently regarded as the first manifestation of coronary heart disease [12]. Since sudden and unexpected death, especially in young people, often lead to forensic autopsy, occasionally cases of very rare diseases are discovered. These include, for example, undetected acquired valve malformations, especially aortic valve stenoses, which represent a higher risk for SCD, vasculitis involving the coronary arteries or a highly stenosing fibromuscular dysplasia of the AV node artery (Fig. 1) [13]. However, the impact of a dysplasia of the AV node artery is still under discussion, as it seems to be more widespread [14]. Acute cardiac deaths with previously unknown amyloidosis of the cardiovascular type with numerous amyloid clods in the myocardium are extremely rare (Fig. 2).

Regardless of age, two major groups of SCD causes can be compared: coronary deaths and non-coronary causes (Tables 1 and 2). Under morphological aspects, a distinction must be made between functionally induced deaths without an apparent morphological correlate and those with a clear and then often typical morphological correlate. Diagnostic difficulties exist particularly in the first group and in cases of minor or atypically pronounced morphological changes in the second group.

**Table 1:** Causes of sudden cardiac death (SCD) in all age groups

Coronary causes	Non-coronary causes
Arteriosclerosis Coronary heart disease (CHD) Myocardial infarction Dissection of arteries Embolism Malformation of the coronary arteries Muscular bridge Coronary aneurysm Arteritis (Fibromuscular dysplasia (FMD))	Heart valve diseases Cardiomyopathy Hypertrophic Dilatative Arrhythmogenic right ventricular [15] Non-compaction Alcoholic Carditis Viral Bacterial Others [16] Hypertonic heart disease Vitium cordis Anomalies of the cardiac conduction system Genetically caused cardiac arrhythmias [17–20] Tumors [21] Other rare causes, e.g. myocardial involvement in amyloidosis [22]

**Table 2:** Frequency of certain causes of SCD in younger adults (according to [23])

Causes of SCD	Frequency (%)
Coronary heart disease	30
Myocarditis, endocarditis	25
Cardiomyopathies	20
Heart valve defect	10
Vitium, cardiovascular anomalies	5
Anomalies of the cardiac conduction system	5
Tumors (mainly myxomas, rhabdomyosarcomas)	1
(Aortic diseases (-rupture))	(4)

Contrary to the cited source, aortic diseases must be separated from SCD

## Coronary heart disease as a cause of sudden death

Baroldi and Fineschi [24] defined the following criteria for the postmortem diagnosis of coronary artery disease and sudden coronary death:

1. Coronary sclerosis of any intensity (including stenosis) but without plaque complications and with normal myocardium does not allow the diagnosis of coronary heart disease (CHD) or SCD.
2. Simple plaques in combination with extensive myocardial fibrosis but without acute myolysis and clinical symptoms justify the diagnosis of chronic but inactive CHD. This form is not a cause of sudden death.
3. The detection of coronary plaques and contraction ligament necrosis of different ages in more than 9 + 6 herds and 102 + 143 necrotic myocytes per 100 mm^2^ indicate acute coronary death.
4. Complicated plaques in normal myocardium or in combination with myocardial fibrosis are compatible with coronary artery disease or a likely SCD.
5. Intimate bleeding alone or in combination with a wall and/or obliterating thrombus in an artery with or without fibrosis in the dependent myocardium makes fresh myocardial infarction (< 1 h) probable; in combination with contraction ligament necrosis, acute myocardial infarction and SCD can be safely considered.
6. The detection of a fresh demarcated infarct zone with or without pronounced plaques justifies the diagnosis of sudden death in myocardial infarction.

The terms ‘complicated’ or ‘active plaque’ are defined as plaque with neuritis, intimata-bleeding or thrombosis or after rupture. Caution should be exercised with regard to the evaluation of contraction bands (of the myocytes), when resuscitation procedures have been performed and catecholamines have been administered [25]. In the event of coronary thrombosis, the age of the thrombus may provide information on the duration of the existing circulatory disorder.

## Acute myocardial infarction and SCD

Death due to acute myocardial infarction (AMI) has been described extensively in the literature, so that a detailed rendition was omitted at this point. The pathophysiological basis of AMI in almost all cases is coronary sclerosis, which can lead either directly to high-grade coronary stenosis or to coronary occlusion based on plaque rupture with secondary thrombosis. Absence of sufficient collaterals results in a reduced perfusion and oxygenation, followed by hypokinesis of the myocardium and finally necrosis in the dependent current area. In about 35 to 85% of cases of AMI, coronary thrombosis can be diagnosed, mainly after plaque rupture [26]. Frequent complications of AMI are arrhythmias, cardiogenic shock, fibrinous epi- and pericarditis, ventricular rupture, aneurysm formation, papillary muscle dysfunction, endocardial thrombus formation and sudden death. The clinical presentation can, but does not necessarily have to, be typical [12].

In addition to cardiac morphology, which exhibits a comparatively typical time-dependent metamorphosis (Table 3), histological and immunohistochemical examination of the tissue and any thrombi may also contribute to the determination of the age of the infarct [27–29].

**Table 3:** Time course of histological changes in the infarction zone within the first 24 h (modified from [1])

Time	Microscopic findings
15 min and more	Increasing distance of the cross stripe in the infarction zone
Up to 30 min	Swelling of the mitochondria and dissolution of the christae
30–60 min	Edema of myocytes, loss of glycogen, contraction ligament necrosis, reduced stainability of myoglobin in immunohistochemistry (IHC), first fibrinogen detection
2–3 h	First hyalinised myocytes in the periphery of the infarct zone, lie staining: dark red ischemic myocytes, positive green fluorescence of the damaged myocardium during fluorochromination with akridine-orange
3–4 h	First agglutinated sarcoleptic tubes, discrete fatty degeneration of myocytes, possible onset of hemorrhagic demarcation
4–5 h	IHC: detection of fibronectin, C5b-9, fibrinogen; depletion of desmin and myoglobin
4–7 h	Necrosis in the infarction zone, first peripheral leukocyte reaction, eosinophilia of the myocytes, shrinkage of the myocytes, loss of nuclear stainability
9 h	Clear necrosis of the infarct zone, strong leukocyte reaction—also in the infarct zone, nuclear staining no longer possible
18–24 h	Extended necrosis zone, significant leukocyte infiltration

## Age determination of thrombi (modified according to Irninger [30])

Irrespective of the causes of thrombus formation, the organism reacts to a thrombus with wound healing processes that are presumably triggered by degradation of the protein and protein structures of the cellular and fibrin-containing components contained in the clot (coagulation necrosis). The endothelialisation of the thrombus surface begins on the first day; after 3 days, fibrin and erythrocytes are completely homogenised (Table 4). The actual organisation begins with the injection of capillaries and the formation of granulation tissue with myofibroblasts and histiocytes. These capillaries are connected to the vascular system and support the recanalisation of the thrombotically occluded vessel. Parts of the thrombus that cannot be recanalised are transformed into connective tissue or myxoid. The result is intimasclerosis or subtotal/total scarring of the vessel.

**Table 4:** Temporal course of thrombus aging (modified after Irninger) [30, 31]

Phase I: 2nd day (1st–3rd day)	Between vascular endothelium and thrombus, there is no reaction. Leukocytes and fibrin strips with thrombocytes unchanged. Erythrocytes mostly densely packed centrally, peripherally looser.
Phase II: 5th day (3rd–8th day)	First endothelial sprouts. Free thrombus surface may have endothelium. Beginning hyalinisation, mostly central. Enclosed leukocytes pycnotic. Monocyte nuclei enlarged and brightened. Shrinkage of the thrombus can lead to crevices and ‘sinuos’ cavities, loosely clenched erythrocytes.
Phase III: 10th day (4th–20th day)	First capillaries, fibroblasts, mesenchymal cells, hemosiderin-storing histiocytes, endothelium under thrombus—hyalinised thrombus divided into larger clods. Isolated nuclear debris of leukocytes. Still distinct myocyte swelling.
Phase IV: 3rd–4th week (8 days to 2 months)	Beginning of fibroplasia (argyrophilic and collagen fibres). Numerous capillaries. Shadowy nuclear debris of leukocytes in the hyalinised thrombus. No more monocyte swelling after the 8th–17th day.
Phase V: 6th month (2nd–8th month)	In addition to a few cell elements, there are isolated capillaries, argyrophilic and collagen fibres, and elastic fibres. Thrombus completely hyalinised, possibly containing cholesterol crystals. Rarely vascularisation from adventitia. Sinusoidal spaces through which blood may have flowed centrally, possibly still thrombus remains.
Phase VI: older than 6–12 months	Completely recanalised by larger vessels, in between tight fibrous and cell-poor connective tissue. Remains of thrombus are missing.

The perifocal inflammatory reaction—with or without bacterial colonisation—leads to secondary loosening of the thrombus. In particular, immigrated granulocytes release proteases, which induce the lysis reaction.

Janssen [32] recommends to examine at least 3- to 6-vessel cross-sections including vessel wall for the histological age determination of thrombi using different staining techniques (HE, van Gieson, Berlin Blue). In the case of embolism, the red tail thrombus should also be examined by longitudinal section. For the question of the minimum or maximum age of a thrombosis, only three stages (1st to 7th day, 5th day to 8th week and older than 2 months) can be differentiated with the certainty required in forensic cases [33].

More recently, attempts have been made to contribute to the determination of thrombus age using immunohistochemistry. The antibodies matrix metalloproteinase-2 (MMP-2), matrix metalloproteinase-9 (MMP-9), urokinase-type plasminogen activator (uPA), tissue-type plasminogen activator (tPA) and plasminogen-activator inhibitor type-1 (PAI-1) proved to be promising [34, 35].

## Myocarditis and SCD

**Fig. 3:**
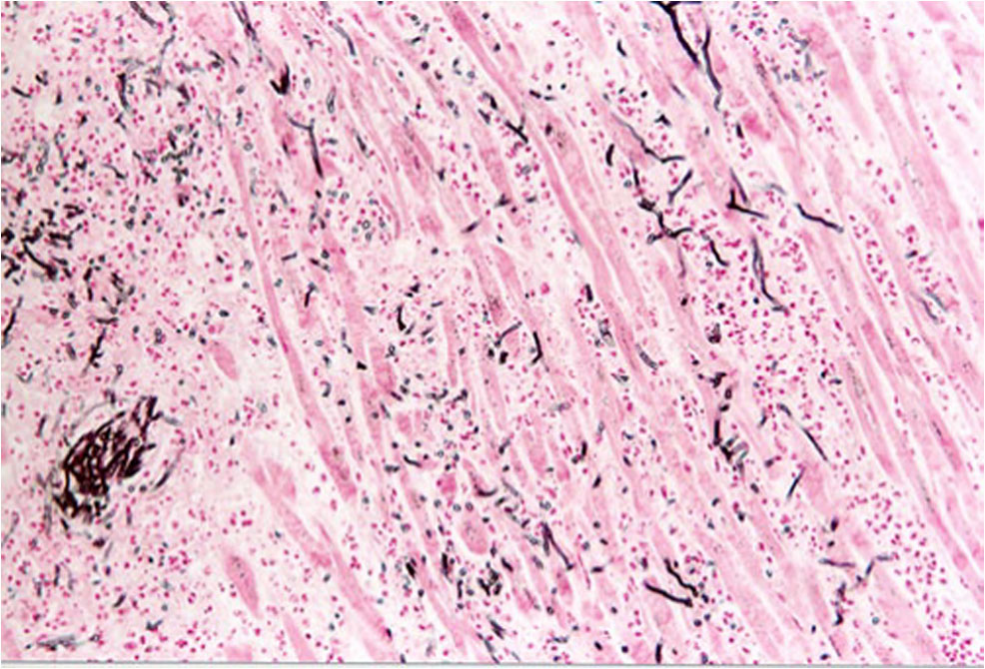
Focal infiltration of the myocardium with fungal filaments and fungal conidia in fungal myocarditis with myocardial necroses and accompanying partial lymphomonocytic, partly also granulocytic infiltration (Grocott staining, × 200)

**Fig. 4:**
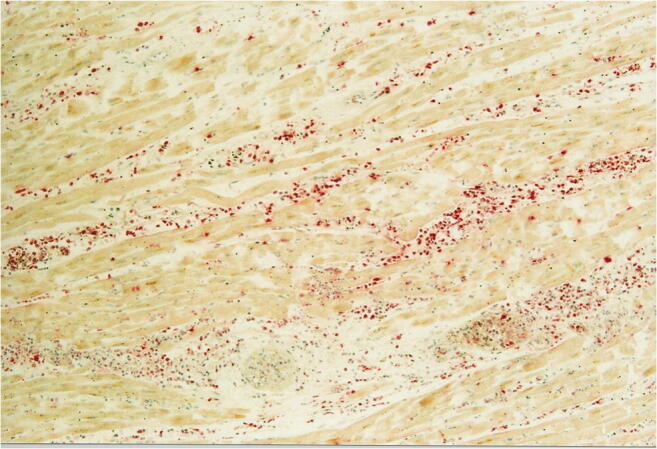
Purulent myocarditis (ASD staining, × 100). Abscess of decaying neutrophil granulocytes and detritus

**Fig. 5:**
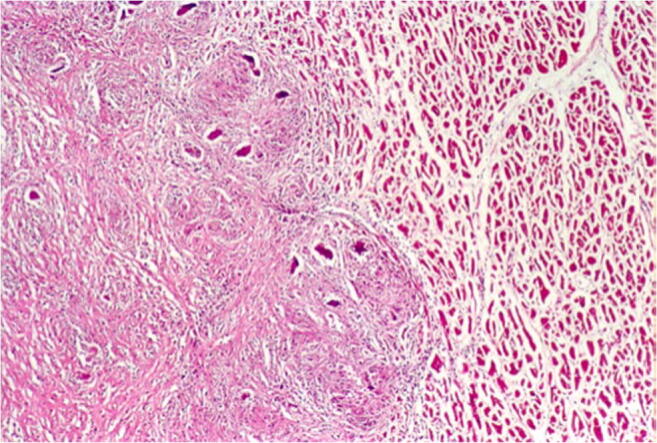
Focal and well-demarcated granulomatous infiltration of the myocardium with multinucleated giant cells, dense fibrosis, and accompanying lymphomonocytic infiltration without necrosis zones (haematoxylin–eosin staining (HE), × 100)

**Fig. 6:**
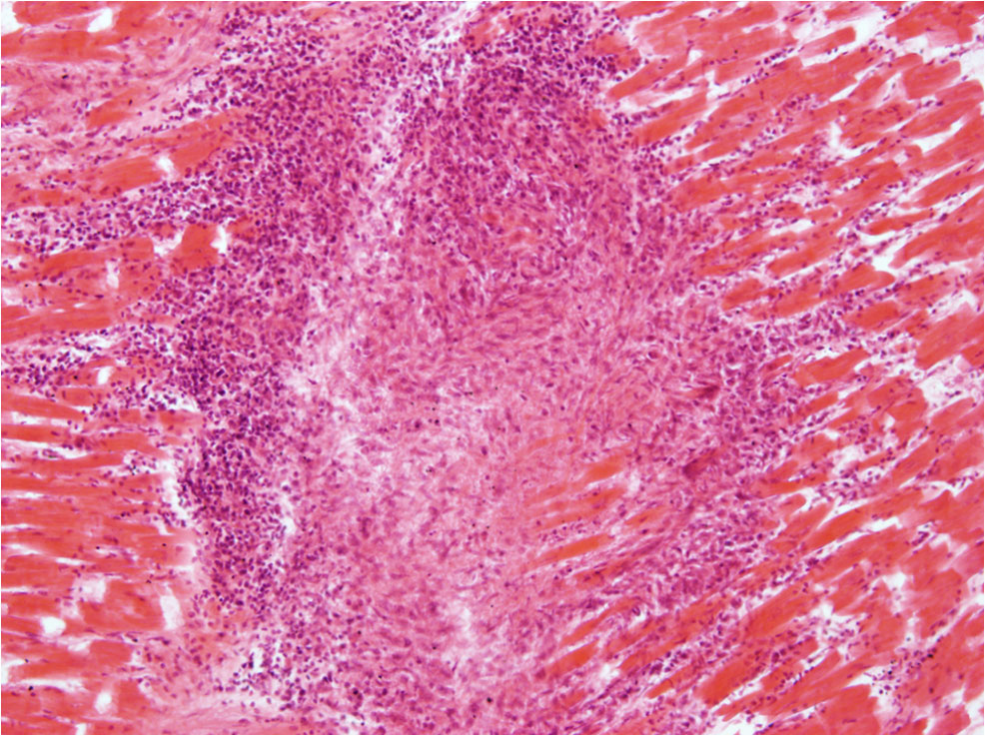
Rheumatoid myocarditis (HE × 200). Aschoff body with granulomatous structures consisting of fibrinoid change, lymphocytic infiltration surrounding necrotic centre

While the diagnosis of myocarditis in living patients is predicted on multiple examinations and is partly biopsy-based, which is defined in the Dallas criteria, as well as immunohistochemical examinations [36, 37], the entire heart is available for examination after autopsy. Thus, all essential regions of the left and right ventricle, the septum, interventricular and atria can be generously included in the histological examination. The aim of diagnostics is the determination of the etiology of myocarditis in addition to the detection of inflammatory infiltrates and myocytolysis. Therefore, bacteriological and virological examinations are just as necessary as the analysis of possible immune complexes (Table 5). While viral myocarditis is the predominant cause of death and bacterial myocarditis occurs during sepsis, fungal myocarditis is rare (Figs. 3 and 4). Occasionally, cardiac sarcoidosis is reported (Fig. 5) [38], tuberculosis and rheumatoid myocarditis are extremely rare as causes of death (Fig. 6). Numerous viruses can be considered as pathogens of viral myocarditisin adults as well as in children, especially enteroviruses, e.g. Coxsackie viruses of group B, are considered to be particularly cardiotropic [39, 40]. Currently, there are also indications that viral lymphocytic myocarditis can occur in the course of COVID-19 infection [41]. A recommendation regarding the diagnostic procedure for suspected acute myocarditis is given in Table 6.

**Table 5:** Etiology of myocarditis (m)

Etiology	Triggering cause
Infection	Bacteria
	Viruses (mainly cardiotropic viruses)
	Fungi
	Protozoa
Allergy	(Auto)immune reactions: eosinophilic (m) and rheumatic (m)
Pharmacotoxic	Drugs
Systemic disease	For example, lupus erythematosus
Physically	Condition after radiotherapy

**Table 6:** Recommended examination methods for the diagnosis of myocarditis (modified from [1])

Diagnostic method	Details
Histology	Fixation: buffered 4% formalin (pH = 7.0), maximum 24–48 h Routine: HE staining of representative samples of all important organs Heart: initially at least 8 samples from different localisations; HE, mallory, LFB, EvG
Immunohistochemistry	Characterisation and quantification of interstitial leukocytes (granulocytes, T lymphocytes, macrophages): e.g. LCA, CD45R0, CD68, CD3; determination of cell count per visual field (× 400) or mm^2^ in up to 20 visual fields and determination of the mean value Detection of proinflammatory proteins and molecules (semi-quantitative): e.g. MHC classes I and II, selectin, cytokines, necrosis markers such as fibronectin, C5b-9, ICAM-1
Molecular genetic diagnostics	PCR and rt-PCR for the detection of viral DNA/RNA: enteroviruses, coxsackieviruses, adenoviruses, epstein-barr virus, parvovirus B19, herpes simplex virus type 6, cytomegalovirus; tissue samples with cellular infiltration should be examined preferably

With regard to the quality and quantity of inflammatory cells, mean pathological values are > 2.0 T lymphocytes (CD3, CD45R0) per field of view (FOV) (× 400) or > 7.0 cells/mm^2^, as well as > 14 T lymphocytes and macrophages (CD68) per visual field—each after counting 20 visual fields and averaging. Additionally, proinflammatory markers, such as MHC class I or II molecules and adhaesion molecules (CD18, CD 54, VLA-4) on cells, should be detected as well at the vascular endothelium [42]. The diagnosis ‘myocarditis’ is considered highly probable, if these cell counts are exceeded.

If microscopically isolated heart-shaped inflammatory infiltrates without myocytolysis are detected, the diagnosis of myocarditis should be made with caution. In these cases, a significant increase in the number of samples and sections is recommended. If microscopically small myocardial infiltrates are found repeatedly, this may justify the diagnosis of myocarditis. Even a few focal inflammatory infiltrates are diagnosed as viral myocarditis, if the molecular detection of (cardiotropic) viruses is also successful. In the case of diffuse myocarditis with extensive myocytolysis, death based on acute heart failure can occur with significant interstitial oedema or damage to adrenergic nerves with subsequent ventricular fibrillation [43]. If other causes of death can be ruled out, rhythmogenic death via an affection of the cardiac conduction system is assumed even in the case of only minor cellular infiltration of the myocardium, but a proven viral genome [44]. Investigations should include nerve fibres with potentially lymphomonocytic infiltration of the perineural nerve sheaths near the cardiac conduction system in cases of SCD [1, 45].

## Genetic causes of SCD

The prevalence of cardiovascular diseases (CVD) within the general population ranges between 3 and 4% [46]. Especially among younger people, predominant causes are structural heart diseases such as cardiomyopathies (CMP) and arrhythmogenic disorders which can partly be attributed to underlying genetic causes [11]. Some of those diseases, for instance different kinds of CMP, present with distinct morphological features that can be detected during autopsy or histological examinations, but in up to 30 % of all SCD cases cause of death cannot be conclusively diagnosed even after autopsy and subsequent examinations (histology, toxicology and biochemistry) [46]. Those cases are referred to as sudden arrhythmogenic deaths (SAD). In cases of SAD, genetic examination, so-called molecular autopsy, could help to determine the cause of death [47].

In recent years, 49 genes could be identified that are associated with important causes of SCD-CMP, CHD and primary arrhythmia syndromes [48].

New developments especially the increased use of next-generation sequencing (NGS) have detected hundreds of mutations within these genes.

Some CVD conditions attributed to changes in one particular gene (monogenetic) can be diagnosed with high probability by genetic testing (like Marfan syndrome), while the sensitivity of genetic testing is quite low for other monogenetic diseases like DCM [46]. Many mutations found so far showed incomplete penetrance, so that it remains unclear if a functional disorder is associated with a proven mutation in individual cases and whether this disorder can be regarded as causing death. Especially for diseases with polygenic background such as CHD, it is even more difficult, if not impossible to reach a conclusive diagnosis by genetic investigations alone.

The diagnostic yield of genetic testing for CVD is highly dependent on clinical presentation of the patient, individual medical history and family history [46, 49]. Unfortunately, in most SCD/SAD cases (within a forensic setting), this information is scarcely available, making it difficult to interpret results of genetic analysis.

Most current studies regarding SAD are based on the guidelines of the American Heart Rhythm Society (HRS) or the European HRS (EHRS), focusing on direct sequencing of the four genes associated with Long QT Syndromes 1–3 (LQTS 1–3), Brugada Syndrome (BrS) and Catecholaminergic Polymorphic Ventricular Tachycardia (CPVT). Those studies yield pathogenic mutations (considered cause of death) in 15 to 20% of the examined cases [50]. In one study, family screening had been performed in addition to postmortal genetic testing thus increasing the diagnostic yield from 26 to 39% [51].

Recently, molecular diagnostics have started to focus on rare variants within genes associated with CVD. As those can occur in patients with or without symptoms, it is challenging to determine which variant is in fact disease causing. The American College of Medical Genetics and Genomics (ACMG) and the Association of Medical Pathologists (AMP) have released a joint statement on the interpretation and classification of variants as being likely pathogenic/pathogenic, likely benign/benign or of uncertain significance. Simultaneously, International Clinical Genome Research working groups are constantly evaluating variants in clinically relevant genes and whether or not a gene is robustly associated with a certain phenotype [52].

For polygenic diseases, thousands of variants can each provide a small increase of risk of disease. A polygenic risk score of the predicted additive effects of those variants can be used to predict the risk of developing a CVD [53].

E/HRS guidelines recommend saving blood and/or tissue samples in all cases of SCD as well as in cases of SAD that occurred within a perceived trigger situation [50]. Those include physical activity, especially swimming for LQT1 [54]. The significance of post mortem genetic testing is not only proof of death, but also the prevention of surviving relatives.

## SCD for athletes

Although the positive effects of regular sporting activity are undisputed, sudden deaths occur repeatedly during physical activities. The incidence of sudden death is estimated at 1.3 to 6.5 per 100,000 people younger than 35 years/year [11, 55]. For the SCD in young athletes, different incidences are found in different populations: there is a SCD incidence of 0.5/100,000 persons in US high school athletes aged between 12 and 24 years [56], while the incidence for Italian athletes (14 to 35 years) was 3.6/100,000 [57]. Thus, the incidence of SCD in young athletes is greater than in young non-athletes. The relative risk for young athletes compared to non-athletes was calculated with an odds ratio (OR) of 2.8 [58]. However, not exercise itself is considered a risk, but rather a combination of intense physical exertion and a pre-existing (possibly unknown) heart disease. Regarding different sports, basketball and football are a particularly high risk [59, 60]. There are also clear gender differences, since the risk is more than twice as high for men than for woman (male: 2.62, female: 1.07/100,000 athletes [55]). An influence of doping is suspected particularly with regard to those cases of SCD for which a cause cannot be determined [61]. The most frequent cause of death is a pre-existing, mostly congenital heart disease, such as cardiomyopathy, coronary artery anomaly or a genetically determined cardiac arrhythmia [55]. For athletes older than 35 years, coronary heart disease and its consequences are the most important. A comprehensive and up-to-date overview of SCD in athletes can be found in Borjesson and Pelliccia [55]. Occasionally, SCD cases occur in athletes with autoptically detectable significant myocardial hypertrophy after many years of taking anabolic steroids [62].

## SCD induced by external forces

Stress has long been considered a risk factor for acute myocardial infarction and SCD [63–65]. Both psychological and physical triggers are mentioned. More frequent psychological causes include death or serious illness of close relatives, financial losses, criminal offences, involvement in traffic accidents (without significant injuries) and visits to the doctor (dentist). Physical stress occurs, for example, during physical activity, sports, especially swimming, sexual intercourse, under the influence of alcohol and drugs and is also triggered by physical pain [66]. Stress should also be able to trigger a special form of cardiomyopathy, Takotsubo cardiomyopathy or ‘broken heart syndrome’ [67]. The disease manifests itself with symptoms similar to an acute myocardial infarction, with temporary ventricular dysfunction, ECG changes and slight increase in heart enzymes, but without any significant coronary heart disease. Specific histological changes are not associated with Takotsubo cardiomyopathy. Histologically, lympho-monocytic infiltrates, macrophages and contraction band necrosis without myocytolysis can be observed [68]. Based on the facts that mainly women in menopause are affected and contraction band necrosis is considered pathognomonic for cardiac adrenergic stress, such a pathomechanism is assumed to be the cause [69]. An overview of the concept of adrenergic stress can be found at Baroldi [70].

Thorax trauma of all kinds are relatively common in forensic practice. Apart from injuries of the lungs, injuries of the large vessels and the heart determine the prognosis. The injury pattern ranges from commotio and contusio cordis to ruptures of vessels and ventricles in blunt trauma or perforating injuries in sharp force or gunshot. Blunt, non-penetrating thoracic trauma acts on the heart via compression of the chest. When falling from height or in certain forms of traffic accidents, injuries are caused by abrupt, sudden delay of the body in filled ventricles with so-called deceleration trauma (pulling effect of the heart on the large vessels with rupture). Finally, chest compression may also lead to a sudden increase of ventricular pressure with subsequent rupture. In addition, there are reports on cardiac concussion, including fatal and non-fatal cases. Immunohistochemically, focal loss of myocardial myoglobin, creatine kinase BB and kreatin kinase MM was identified with scattered deposition of these substances between myocardial fibres elsewhere in canine models [1, 71–73].

The evaluation of SCD is particularly difficult, if the consequences of cardiac injury are not immediately obvious. In this case, one must think of commotio cordis, which can characteristically lead to death with subsequent rhythm disorder, usually ventricular fibrillation. In about 60% of cases, death occurs immediately, while for the remaining 40%, a short survival time is described [70]. Typical for SCD in commotio cordis is a blunt force action against the anterior lower thoracic region (precordial), which is supposed to hit the heart in vulnerable period of repolarisation (in front of the T-wave of the ECG). Although, by definition, morphological changes in commotio cordis should not be detectable, microscopic muscle cell damage is described at least in animal experiments: these are widened I-bands and hypercontraction with contraction band necrosis [74]. Furthermore, an increased CK-MB concentration was described [75]. In the case of violent effects against previously damaged vessels, vascular injuries are conceivable which, for example, can lead to trauma-induced ruptured plaques with subsequent thrombus formation and thus to acute vascular occlusion. Finally, blunt thoracic trauma can also be associated with various forms of pericarditis (serofibrinous, chronic constrictive) [76]. The literature is controversial on the possibly fatal threat posed by electro weapons (‘tasers’), e.g. in cardiac patients or pacemaker wearers; however, corresponding deaths have been repeatedly reported [77, 78].

Heart catheter examinations are often performed for different indications. In coronary angiographies, balloon dilatation of the coronary clearings and the insertion of stents, an acute coronary thrombosis or a (initially covered) coronary wall rupture with subsequent acute lethal pericardial tamponade can rarely occur [79]. For more than 9000 pulmonary catheter examinations, Procaccini and Clementi report various complications (defective vascular puncture, pneumothorax, series of ventricular extrasystoles, atrial fibrillation) in less than 3% (N = 275) of patients [80]. Among these were only 7 cases with vascular wall or heart rupture. In electroablation for cardiac arrhythmias, perforation of the heart wall at atrial level is a rare but typical risk [81].

Complications of cardiopulmonary resuscitation procedures are well known and may also affect the heart [82]. However, these severe complications such as pericardial and myocardial injuries due to fractured ribs, ventricular or atrial ruptures with development of a hemopericardium are very rare [83]. Of course, the examiner’s task in such cases is not only to prove the complication of the resuscitation procedures but also to clarify the disease leading to it.

### Effects of medication and drugs

Secondary cardiomyopathies, myocarditis, the large group of cardiac arrhythmias and SCD may be caused by legal or illegal drugs.

Alcoholic cardiomyopathy belongs to the dilated forms of the disease [84, 85]. It is believed to be induced by a toxic effect of alcohol. Histologically, hypertrophic cardiomyocytes with different core sizes dominate. Interstitial and endocardial fibrosis can occur as well as fibre ruptures; empty sarcolema tubes can be observed. Single cell necrosis can also be the cause of macrophage infiltration. This diagnosis cannot be derived from the examination of the heart alone. The differential diagnosis is mainly chronic inflammatory cardiomyopathy (DCMi) [86]. The anamnesis often gives indications of chronic alcohol abuse and thus of a possible disease. In addition, other alcohol-associated changes, especially of the liver and pancreas, must be taken into account [1].

**Fig. 7:**
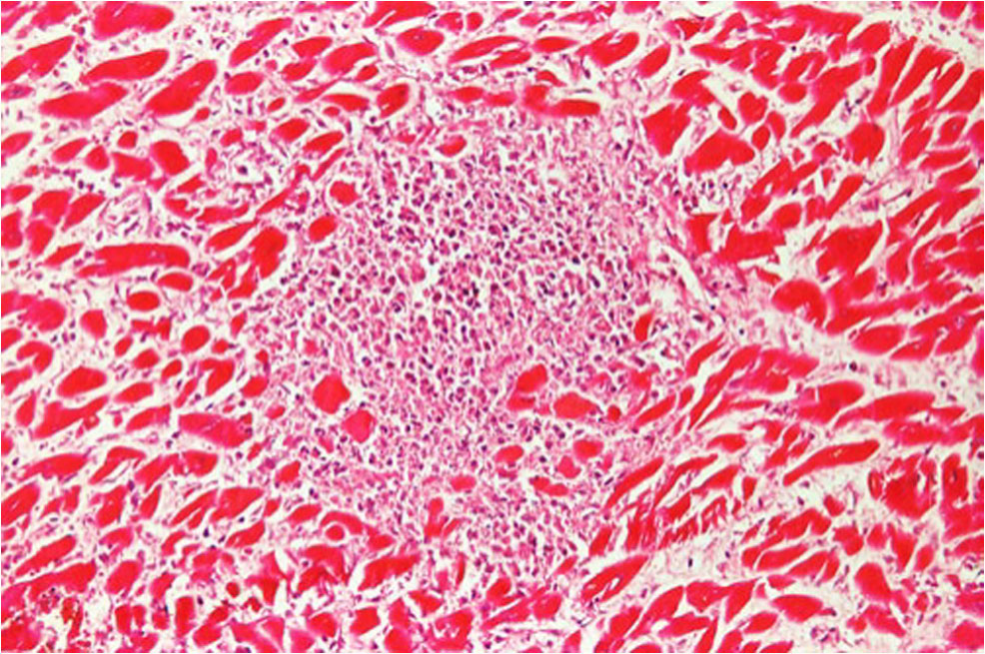
Good demarcated necroses in the myocardium with dense collections of lymphocytes and monocytes, isolated plasma cells and eosinophil granulocytes for diclofenac myocarditis (HE staining, × 200)

Myocarditis can have allergic or pharmacotoxic causes [1]. These forms of the disease are often characterised by eosinophilic granulocytes, lymphomonocytic inflates and single cell necrosis (Fig. 7). Cardiomyocytes are occasionally characterised by a homogeneous eosinophilic cytoplasm. However, the histomorphology is diverse. Triggers can be anticonvulsants, neuroleptics and diuretics. Clozapine myocarditis is particularly well known [87].

The large number of drugs that cause cardiac arrhythmias as side effects or because of taking overtherapeutic doses (intoxication) and/or also reduced excretion is only marginally mentioned. The best known and probably also most frequent example in older people is digitoxin/digoxin, which can lead to bradycardic cardiac arrhythmias in older people with existing renal insufficiency due to its small therapeutic width and renal excretion. Here, the cause is occasionally overlooked despite typical symptoms [88].

Among illegal drugs with cardiac effects, cocaine and amphetamines (MDMA—ecstasy, MDEA—Eve) are particularly noteworthy. Cocaine acts as a strong sympathomimetic and can induce hypertension and arteriosclerosis, spasms of the coronary arteries, myocardial ischemia up to the so-called cocaine cardiomyopathy, or trigger a myocardial infarction as well as cardiac arrhythmia with ventricular fibrillation [89, 90]. The toxic effects of MDMA and MDEA are not dose-dependent. The risk of sudden death for first-time users is given as 1:2000 to 1:50,000 [91]. In the cardiovascular system, the intake can lead to hypertension and tachycardia. An association with acute myocardial infarction is also described [92, 93]. In addition, rhabdomyolysis and disseminated intravascular coagulation disorders are more frequent findings.

Inflammatory diseases of the myocardium and/or heart valves result particularly from infections caused by intravenous drug use and contamination of the syringe set. They occasionally cause sudden death of drug abusers.

## Diagnostic procedure if SCD is suspected

The Association for European Cardiovascular Pathologists has been published guidelines for the investigation of SCD in 2008, updated in 2017 [94, 95]. In this document, the role of autopsy is defined as follows:

The autopsy should investigate

1. Whether death is attributable to a cardiac disease or to other causes of sudden death (SD);
2. The nature of the cardiac disease and whether the mechanism was arrhythmic or mechanical;
3. Whether the condition causing SD may be inherited, requiring screening and counseling of the next of kin
4. The possibility of toxic or illicit drug abuse, trauma and other unnatural causes;
5. The role of third persons in the death.

The procedure for any examination is complex and begins with the anamnesis. All relevant information on the history of the disease, family history, lifestyle, current medication and circumstances of death including possible resuscitation procedures should be collected. The question whether or not sudden death did occur in the family and a new evaluation of any ECG derived earlier can be helpful.

The actual autopsy begins with the usual external examination. Particular attention should be paid to physical features that are more frequently associated with heart diseases (e.g. watch glass nails, drum flail fingers etc.). It should be searched for implanted pacemakers and other electrical devices. These devices should definitely be read out by a cardiologically experienced specialist before the autopsy begins, as this will record indications/documents for the pathomechanism leading to death (rhythm disturbances) that cannot be proven morphologically [96]. If a defibrillator is present, it is recommended to switch it off and explant it before beginning the internal inspection [97]. The internal examination as well as the careful macromorphological examination of the heart serve to exclude extracardiac causes of death. This includes weighing the heart and measuring the circumference of the valves and the thickness of the walls of the ventricles. If the pericardium and epicardium do not have particular characteristics, the outlet of the vessels or the anatomy of the valve apparatus and the atrial and ventricular septa (including Foramen ovale) can be checked. Finally, the coronary arteries must be examined [87] and the tissue samples have to be selected for subsequent histological examination. Basso et al. [94] gave detailed recommendations for this as well giving an overview of the value of the findings during the autopsy (Table 7). The material should be fixed in buffered 4 (− 10%) formalin. Cutting can be performed after 24-h fixation time. It is recommended to fix several small tissue samples (1 × 1 × 1 mm) in 2.5% glutaraldehyde for electron microscopy as early as possible, if it is suspected that death may have occurred due to rare cardiomyopathies.

**Table 7:** Diagnostic certainty of the cause of death at SCD (according to Basso et al. [98])

Sure	Most likely	Doubtfull
Massive pulmonary embolism	Stable plaque with > 75% stenosis without infarct scar	Minor coronary artery anomalies
Hemopericardium in aortic or ventricular rupture	Exit of the left coronary artery from the right sinus	Course of coronary arteries under muscle bridges
Tearing off of a papillary muscle or tendon rupture with mitral valve insufficiency and pulmonary edema	Cardiomyopathy (hypertrophic, dilatative, ARVCM)	Focal myocarditis, hypertonic heart disease, idiopathic left ventricular hypertrophy
Acute coronary occlusion by thrombus, dissection or embolism	Myxoid degeneration of the mitral valve with prolapse, atrial dilatation, and ventricular hypertrophy	Myxoid mitral valve degeneration with prolapse, but without atrial dilatation
Exit of a coronary artery from the pulmonary trunk	ECG-documented excitation disorder (WPW syndrome, Lown-Ganong-Levine syndrome)	Calcification of the membranous septum, atrial septum lipoma
Closure of a valve by thrombus or tumor	ECG-documented SA or AV block	AV node tumor without ECG changes, anomaly of the conduction system without ECG changes.
Thrombotic block or removal/removal of a valve prosthesis with insufficiency	Congenital vitium cordis (after surgery)	Vitien—not operated, with and without Eisenmenger syndrome
Massive acute myocarditis		

Samples for toxicological analysis (at least heart blood, thigh vein blood, stomach contents, urine, cerebrospinal fluid) and for ‘molecular pathology’ should also be taken before formalin fixation and stored at 4 − 8° C until qualitative/quantitative examination.

Molecular genetic investigations cover the detection of DNA/RNA of pathogens causing inflammatory diseases and mutation analysis in cases of suspected genetic heart disease. Ten-milliliter EDTA blood and 5 g each of heart tissue and spleen tissue, which can be stored frozen at − 80 °C or in RNA later at 4 °C for up to 2 weeks, are sufficient for the tests [86]. The use of neutrally buffered formalin (4%, pH 7.0) is recommended, if a molecular pathological examination of paraffin-embedded myocardial tissue may be considered. The fixation period should be as short as possible as 24 to 48 h.

Further diagnostic possibilities seem to emerge in the field of imaging procedures. These include postmortem endoscopy (minimally invasive autopsy), postmortem computed tomography (PMCT), postmortem MR (PMRT), postmortem angiography (PMCTA) and postmortem multiphase CT angiography (MPMCTA).

## Conclusion for practical case work

Sudden deaths from natural causes are predominantly due to cardiac causes, in older people often to complications of coronary sclerosis. Especially in younger people and in the absence of coronary sclerosis, functional causes of death should be considered, such as genetic-determined cardiac arrhythmias or inflammatory diseases. Their clarification requires a higher diagnostic effort with inclusion of molecular genetic investigations for the detection of pathogens or mutations. Here, such a possibility must already be considered during the autopsy, so that suitable examination material is preserved and stored.
